# 3D digital stereophotogrammetry: a practical guide to facial image acquisition

**DOI:** 10.1186/1746-160X-6-18

**Published:** 2010-07-28

**Authors:** Carrie L Heike, Kristen Upson, Erik Stuhaug, Seth M Weinberg

**Affiliations:** 1 Department of Pediatrics, University of Washington, Seattle, WA, USA; 2 Children's Craniofacial Center, Seattle Children's Hospital, Seattle, WA, USA; 3 Department of Epidemiology, University of Washington, Seattle, WA, USA; 4 Center for Craniofacial and Dental Genetics, University of Pittsburgh, Pittsburgh, PA, USA

## Abstract

The use of 3D surface imaging technology is becoming increasingly common in craniofacial clinics and research centers. Due to fast capture speeds and ease of use, 3D digital stereophotogrammetry is quickly becoming the preferred facial surface imaging modality. These systems can serve as an unparalleled tool for craniofacial surgeons, proving an objective digital archive of the patient's face without exposure to radiation. Acquiring consistent high-quality 3D facial captures requires planning and knowledge of the limitations of these devices. Currently, there are few resources available to help new users of this technology with the challenges they will inevitably confront. To address this deficit, this report will highlight a number of common issues that can interfere with the 3D capture process and offer practical solutions to optimize image quality.

## Introduction

Methods that allow for the objective assessment of facial form are becoming increasingly important for research in dysmorphology, genetics, orthodontics and surgical disciplines among others [1-8]. Such methods also have the potential to enhance clinical care by facilitating surgical planning, improving outcome assessment, and aiding in syndrome delineation [8-13]. Non-contact 3D surface imaging systems are rapidly replacing traditional "hands-on" anthropometry as the preferred method for capturing quantitative information about the facial soft-tissues [14,15]. These systems offer a number of distinct advantages: minimal invasiveness, quick capture speeds (often under one second), and the ability to archive images for subsequent analyses [16,17]. In addition, a number of independent studies have demonstrated a high degree of precision and accuracy across a wide variety of 3D surface platforms [18-30]. The safety, speed and reliability of data acquisition that these systems offer are particularly helpful when working with young children, for whom quantification of facial features can be challenging [31,32].

**Figure 1:**
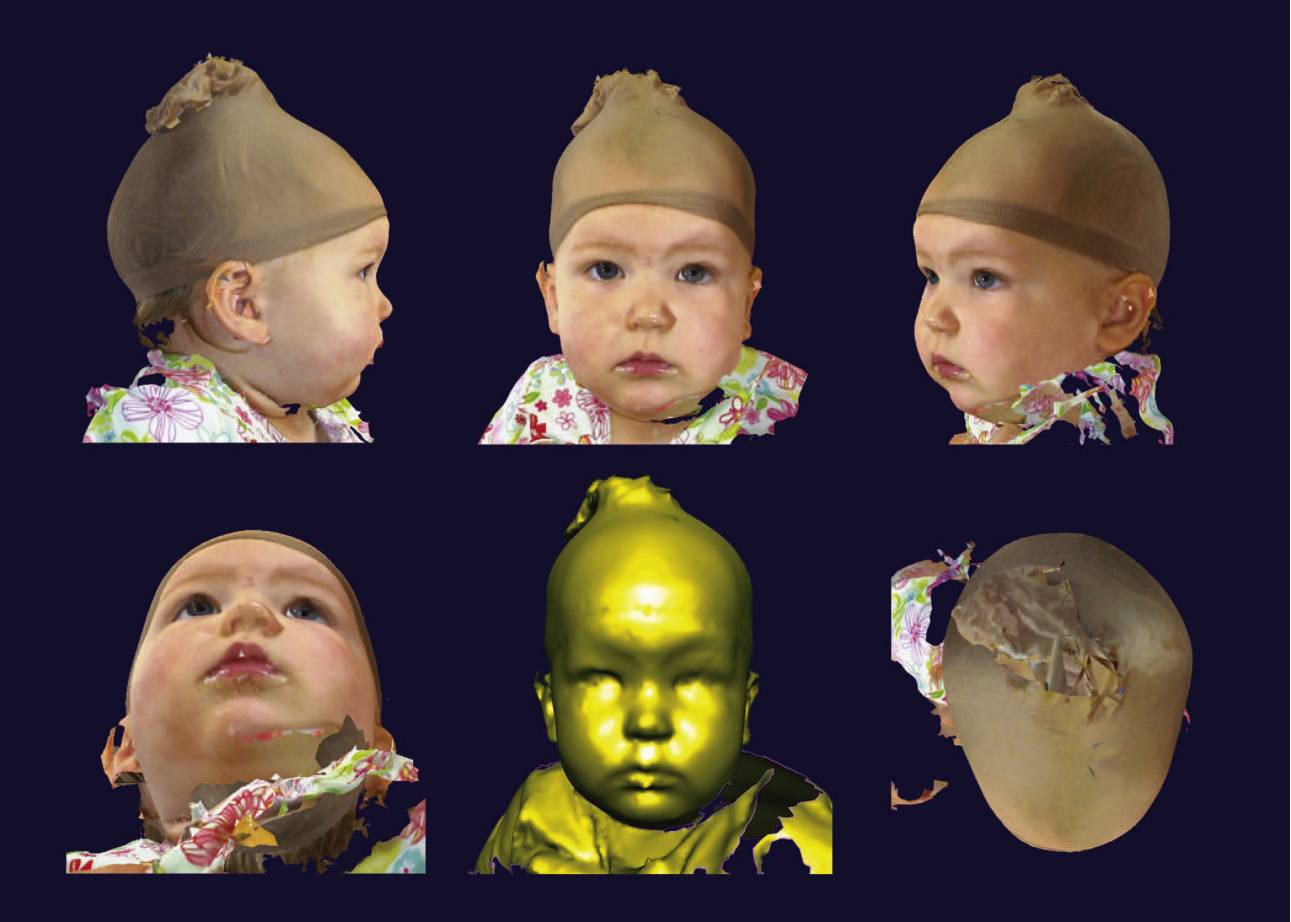
**Example of a two-dimensional screen capture of a 3D facial surface model **The capture is alternatively rendered to show the underlying geometry, as well as color and texture information mapped onto the surface. Written consent for publication of this image was obtained from the participant's parent.

The most common class of 3D surface imaging system is based on digital stereophotogrammetric technology. These systems are capable of accurately reproducing the surface geometry of the face, and map realistic color and texture data onto the geometric shape resulting in a lifelike rendering (Fig. 1). The mathematical and optical engineering principles involved in the creation of 3D photogrammetric surface images have been thoroughly described [16,33-35]. The combination of fast acquisition speed and expanded surface coverage (up to 360 degrees) offer distinct advantages over older surface imaging modalities like laser scanning.

With decreasing cost, 3D stereophotogrammetric imaging systems are becoming increasingly common in clinical and research settings [36,37]. With any new technology, a number of factors must be considered in order to achieve optimal performance. Though camera manufacturers provide suggestions for device set up and calibration, limited information is available on the practical issues that will inevitably confront new users of this technology. However, such issues can adversely impact the reliability of data collection, and consequently, influence the clinical and research study results. In order to ensure optimal interpretation of the study results, all aspects of data collection should be rigorously evaluated [38].

This report will serve to highlight a number of common issues that can interfere with the 3D facial capture process and will offer practical solutions and recommendations to optimize image quality.

## The Imaging Environment

### Location and placement

**Figure 2:**
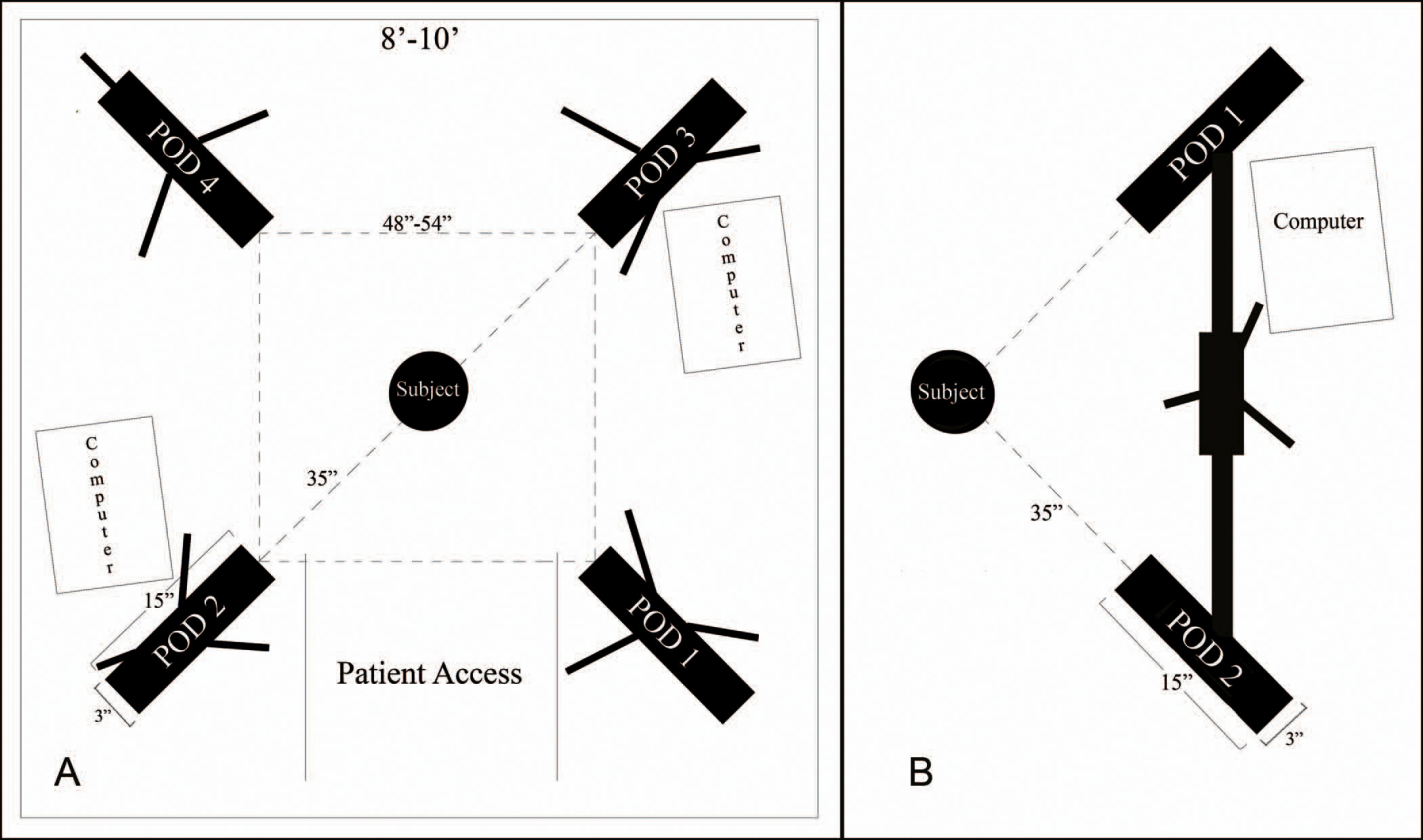
**Illustration showing example floor footprints for two different imaging set-ups **(A) 360 degree image capture system for imaging the entire head and face; (B) 160-180 degree image capture system designed to capture the face.

**Figure 3:**
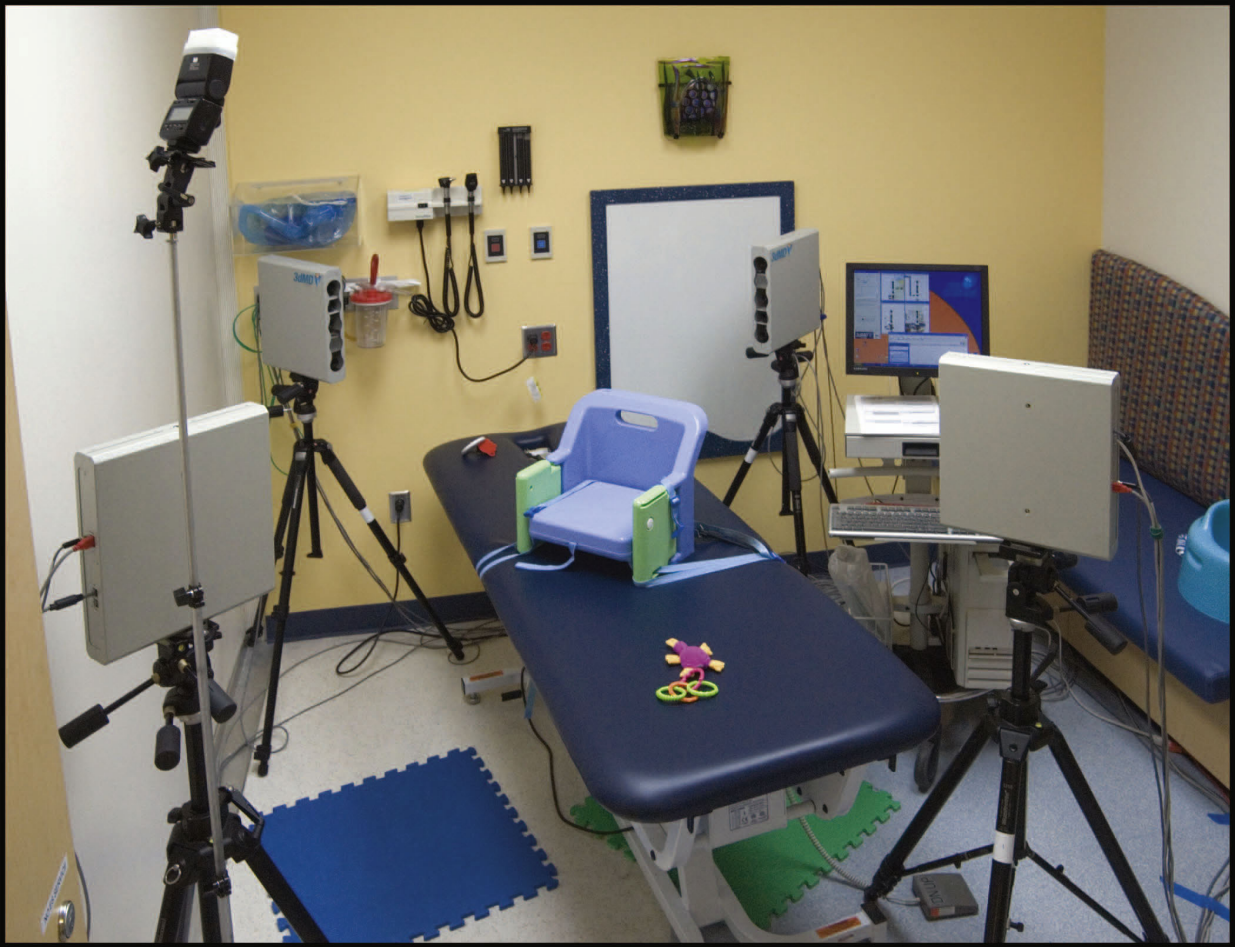
**An example of a 3D stereophotogrammetry system (3dMDcranial™ System) in a clinical research setting **The mechanical bed offers a safe surface upon which to secure a booster seat, while allowing the photographer to adjust the participant to ensure an optimal image capture.

When choosing a location to set up a 3D photogrammetry system, the most essential consideration is space. The minimum space requirements for a given system must account for the major components of the device, which typically include the imaging hardware, a tripod or other mounting system, a computer, a cart or table for the computer and a seat for the subject (Figs. 2 and 3). The space must be adequate to accommodate: the physical footprint of the assembled imaging system, the computer that controls the imaging system, the subject and requisite seating, and pathways for the operator to move about unencumbered during the capture process.

Although practical concerns will often govern placement, factors such as availability of a reliable power source, access to internet and/or network ports, and the flow of foot traffic through the space (particularly if the system is in a public space) should be considered. It is also helpful for the operator to be able to view the computer screen during the capture process.

### Ambient lighting

Different 3D photogrammetry systems have different ambient lighting requirements, but office lighting conditions (e.g. overhead fluorescents) are usually adequate. The adverse influence of suboptimal lighting typically occurs immediately preceding 3D capture, when the cameras display real-time video which allows the operator to adjust the position of the subject for optimal coverage. If the ambient light is too bright or dark, it may overwhelm the camera's sensors during this phase. During image capture, most systems are fairly robust to a range of ambient lighting conditions because they employ their own internal (or external) flash mechanisms [16]. However, excessive light may interfere with the system's flash units. This can occur when the system is set up adjacent to a large window with direct sunlight. If the system cannot be relocated, adjustable window blinds or shades can minimize the effects of sunlight.

### Installation options

Permanent installation may be an option for some 3D systems. The advantages of permanent installation include: reduced wear-and-tear on the equipment, greater consistency in data collection and quality, and time savings. However, if mobility is required or dedicated space is not available, then the system may need to be assembled and disassembled as needed [16]. In this scenario, protective casing can ensure that the sensitive equipment can be stored and transported safely. Hard cases equipped with customizable high-density foam offer such protection.

### Seating options

A variety of seating options will work well for most 3D surface imaging environments. Two criteria to consider include: (1) the ability to adjust the seat's vertical height to accommodate subjects of varying heights and (2) back support to keep subjects in the correct posture. For investigators using a 360-degree view system, it is important to ensure that the chair's back height does not interfere with the image acquisition from rear cameras. For systems where the subject must be positioned to fit within a narrow imaging window, casters allow for multidirectional mobility on most surfaces. Newer digital stereophotogrammetry systems have fast capture speeds that obviate the need for head restraint.

### Safety and security precautions

The 3D imaging environment presents some physical obstacles to subjects and operators. The cables and cords that connect the imaging components, particularly cables that traverse areas of foot traffic, should be bundled. Taping cables to the floor prevents tripping. Tripod legs can also pose a tripping hazard. Allotting enough room to provide an unobstructed route through the imaging environment is essential for participant safety and to avoid the need for recalibration if the camera system is disrupted.

## Maximizing Image Quality

### Reducing artifacts

**Figure 4:**
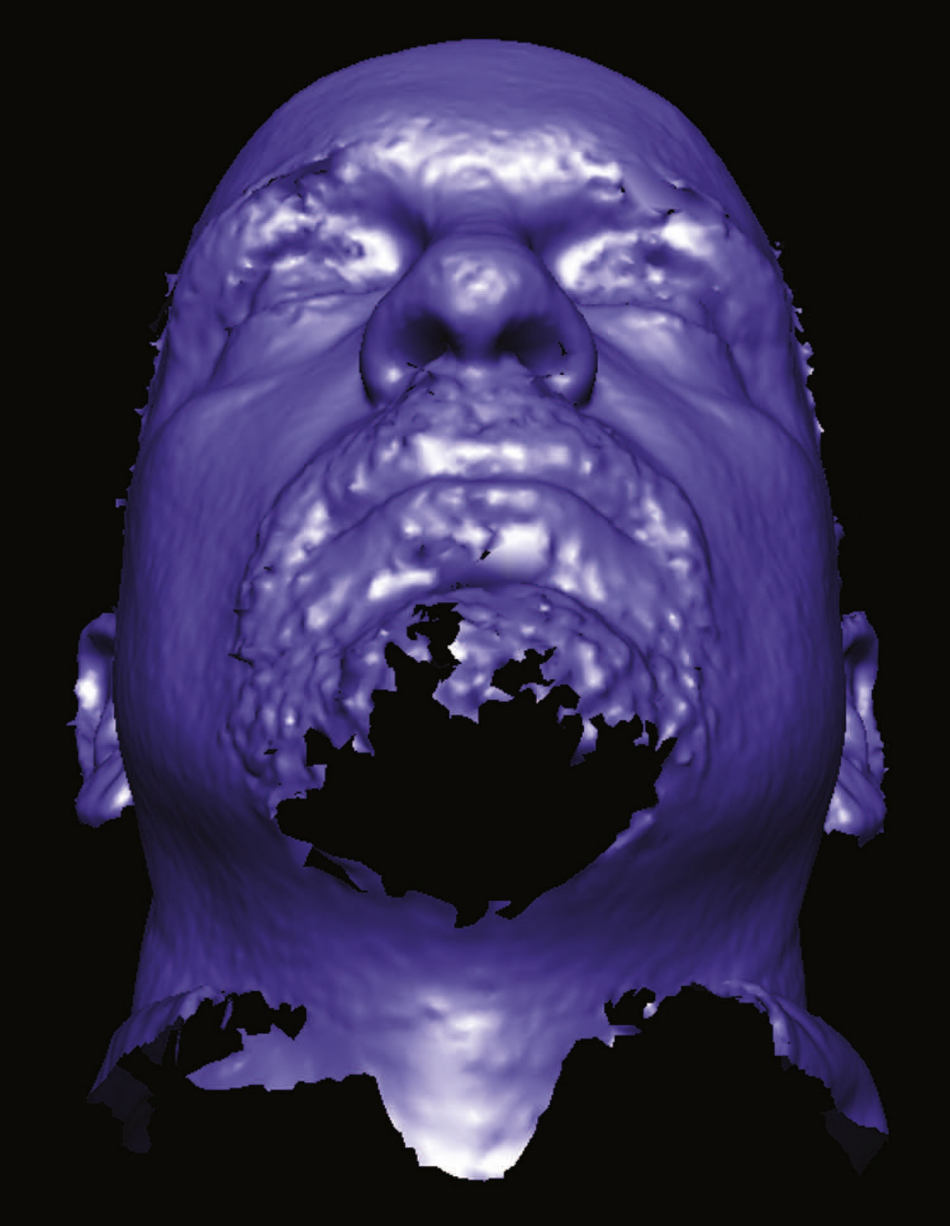
**Surface data loss due to the presence of excess facial hair **Color and texture information have been removed from this 3D model.

**Figure 5:**
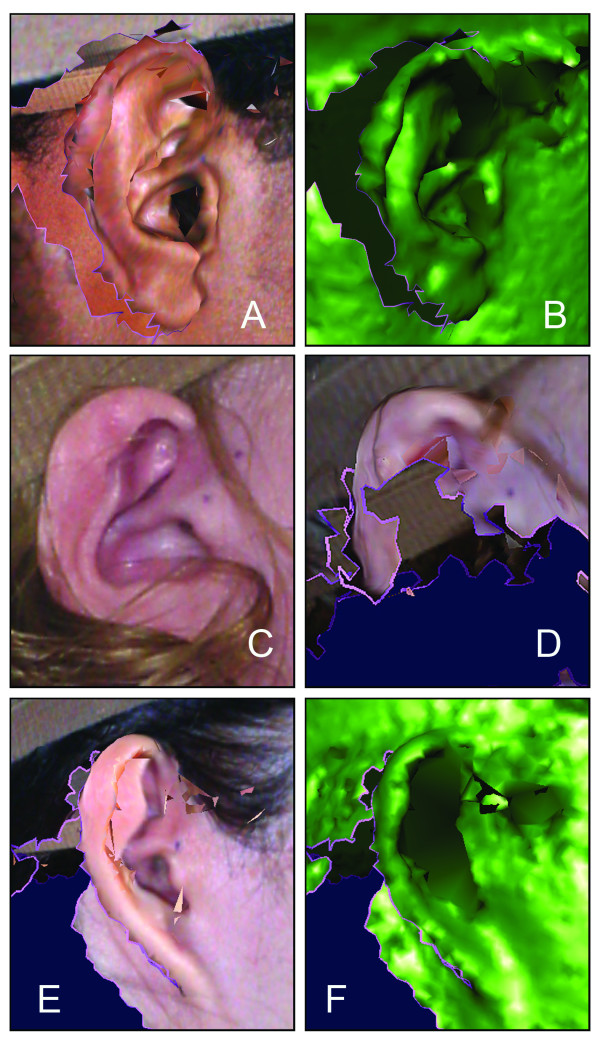
**Example of inadequate surface coverage on the ear **Poor ear coverage may occur due to the angle at which the participant was facing relative to the cameras at the time of image capture (A and B), or due to interference from scalp hair (C and D). Due to the intricacy of the external ear, detailed data beyond height and width may not be attainable for some individuals (E and F).

Most digital stereophotogrammetry systems have difficulty capturing hair, which can result in a substantial loss of surface data on the head and face (Figs. 4 and 5). The forehead and the ears are the regions most vulnerable to interference from scalp hair [16]. Pins, barrettes and hairbands can be effective when used either alone or in combination [24,39,40]. Snug fitting wig caps work well; however, care must be taken to avoid placing excess tension on the skin, which can alter the facial surface [41]. Little can be done to mitigate the effects of facial hair in men.

Surface regions in close proximity to reflective objects (e.g. eyeglasses, earrings, necklaces) are another source of image artifacts. Whenever possible, subjects should remove glasses and jewelry [42,43]. Noserings and other piercings may be too difficult to remove. Likewise, shiny surfaces, primarily due to oily skin or cosmetics, can create artifacts on images [15,28]. A light dusting of powder around the nose, ear and forehead can reduce shininess.

Removal of sweatshirts with hoods, and tucking in collars and other clothing articles around the neckline facilitates adequate capture of the neck, mandible, and ear.

### Achieving a "neutral" facial expression

For most applications, it is ideal to have subjects maintain a neutral facial expression during image capture [43-47]. It is usually sufficient to instruct subjects to relax their face. In addition to obvious signs of facial tension (e.g., furrowed brows) or emotional expressions, operators should pay attention to the subject's mouth and eyes [7,38,48]. An open mouth will artificially extend the vertical height of the face and alter the position of the mandible. To avoid this, the subject's mouth should be closed during capture, with the lips gently pressed together. With the mouth closed, the natural resting jaw position is sufficient in most cases; however, some studies may require that the subject achieve a relaxed dental occlusion [47,49,50]. If image capture of the exocanthion (outer corner of the eye) and endocanthion (inner corner of the eye) are important, then the subject's eyes should be fully open during image aquisition [29]. A visual target helps the subject to fix their gaze in the optimal direction. A mirror may assist participants with achieving the desired position and expression [51]. For younger children, additional steps may be required to achieve a neutral expression (discussed below) [24].

### Ensuring optimal coverage

The most important facial regions to capture will vary according the specific clinical or research question. The imaging technology is usually the limiting factor in how much surface data can be reliably captured in an image, determined in part by the physical distance between the cameras. A single standard frontal 3D capture of the face will produce consistently reliable data from approximately 160 to 180 degrees for many systems. Even in systems capable of true 180-degree capture, ear-to-ear coverage can be poor in a straight frontal capture, particularly in a subject with a very broad upper face [29]. Additional captures may be required (e.g., from the subject's side) to adequately capture both ears [16,41,52]. Some modular systems can be expanded to 360-degree coverage [24]; however, this increases the expense and footprint requirements.

**Figure 6:**
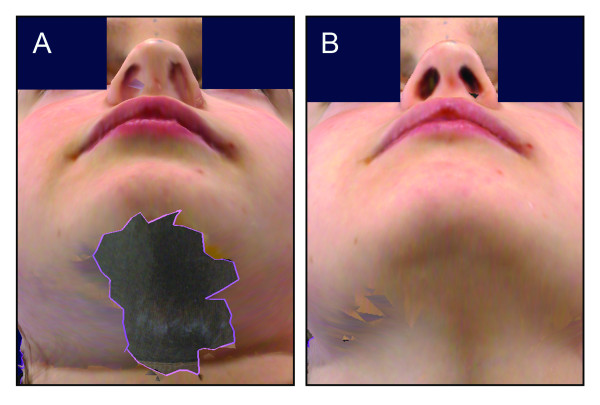
**Example of data loss in the subnasal and submental regions **Poor resolution and data loss (A) may be minimized by tilting the head back (B).

The subnasal and submental regions are prone to data loss and artifact. Proper head positioning can ensure that these regions are visible to the imaging sensors. Titling the subject's head back a few degrees is often sufficient to capture these regions (Fig. 6) [44,53,54]. Vertical adjustment may be necessary to ensure that the subject's entire face is in the imaging frame. This can be accomplished with an adjustable chair and/or an adjustable tripod(s) [51]. If detailed assessment of the subnasal region is required (e.g., with an assessment of nostril shape/asymmetry), the operator can ask the subject to extend the neck and tilt the head back for additional images [55].

### Evaluating the results

Investigators can either preview images at the time of image acquisition or obtain additional images to minimize the possibility of missing data during image acquisition. Reviewing 3D images for key features (Appendix 1) at the time of image capture requires immediate image processing, which may take several minutes. If problems are recognized while the participant is present, then additional captures can be acquired at that time [24].

It may not be feasible to review images at the time of image acquisition, such as when working with large groups. In this case, investigators can acquire multiple images for each participant to maximize the likelihood of obtaining adequate data coverage, and process the images later for subsequent evaluation.

## Working with various populations

### Infants and young children

Working with young children can pose unique challenges [24,36,56,57]. First, it is essential to provide the child and parent with a safe route to the seating area so that they do not disrupt the pods. As toddlers and pre-school children can be unpredictable, it is usually best to ask the parents to hold them until they are securely placed in the chair. The child's anxiety about the equipment is usually tempered by allowing the parent to sit next to or with the child [24,36], so there must be room for the adult to maneuver without disrupting the equipment.

**Figure 7:**
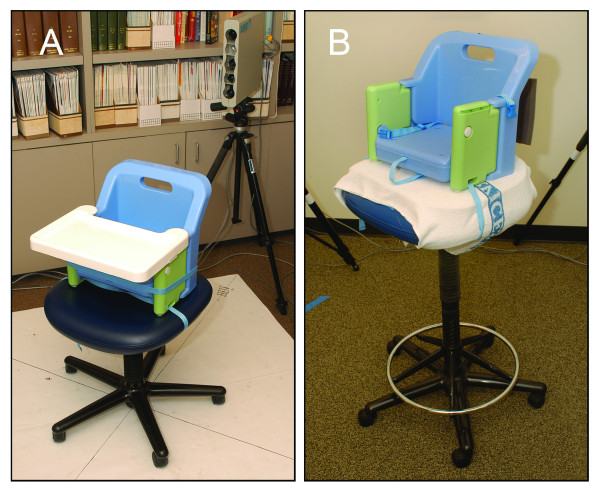
**Seating options for infants and toddlers **These may include booster seats securely strapped to adjustable chairs (A and B). The chair backs have been modified to ensure safety (B). The height range for the chair can be enhanced by the use of additional supports (B).

To maximize patient safety, we recommend that infants and toddlers who are able to sit be placed in a booster seat that is securely strapped to the adjustable chair (ideally with a wide seat). Infants 5-10 months of age who are able to sit with minimal support often do well in a booster chair with moderate support. Infants and toddlers 9 months-3 years of age who are able to sit independently, can be placed in a regular booster chair (Fig. 7). To ensure adequate safety, we recommend that an adult stay near the child during image acquisition.

An adjustable chair saves space and easily fits between the pods; however, some infants and toddlers need to be held by a parent to remain relaxed. Alternatively, a mechanical platform (e.g. clinical exam table) works well (Fig. 3) [40]. These beds are excellent for accommodating parents, and offer a secure seat for children of all ages. However, a larger space is required.

Facial expressions may alter position of landmarks and affect the reliability of facial measurements [57]. It is natural for children to want to 'smile for the camera', which may not be optimal. Older children can follow instructions to keep neutral, relaxed face, with the mouth shut and lips gently touching [58,59]. It may also help to ask them to swallow and relax [29,60]. Younger children often require distraction devices to focus their attention in the preferred direction, and these devices should not elicit facial expressions (e.g., laughter or a surprised look). Such distraction devices include bubbles, toys with soft sounds and/or lights, or a children's video.

Wiping the noses and mouth areas of infants and toddlers just prior to image capture can minimize reflection from wet surfaces that create artifacts.

### Individuals with special needs

The unique considerations for individuals with special needs must be taken into account when developing a 3D imaging protocol [41,61]. It should be anticipated, for example, that some individuals may exhibit inattentiveness, may be overwhelmed by the appearance of the imaging system, may be sensitive to wearing a wig cap, or may be unable to maintain the facial expressions requested for a given clinical or research study. These issues are likely to be present to some degree when working with individuals with mental health conditions [52]. Such factors can present a unique set of challenges for quality image acquisition. It is important to be sensitive to the participant and these potential issues. In these situations, the operator should expect to take multiple repeated captures and factor in the extra time accordingly.

### Large groups

When a large number of individuals need to be imaged in rapid succession (e.g., on-site at medical conferences), it can be challenging to maintain quality control, while maximizing efficiency. Processing each surface can take as long as five minutes, which may not be feasible under field conditions. Therefore, many systems offer a "batch processing" option to allow the operator to capture a series of images rapidly. However, this requires the operator to postpone the image processing step, so inspection of the resulting 3D models while the participants are still present is often not as feasible.

## Conclusion

3D surface imaging technology can serve as a powerful tool to capture and quantify craniofacial morphology. Acquiring high-quality 3D facial images requires methods to optimize the image capture process. Our goal was to provide the reader with a review of the common issues likely to confront users of this technology, refer readers to additional studies which have acknowledged these factors, and provide practical solutions. We summarize some general recommendations to optimize 3D facial image acquisition in Appendix 2. It is up to the reader to determine the applicability of the aforementioned techniques to their specific research or clinical question.

## Appendix 1. Questions to consider when reviewing 3D images

• Is the subject's facial expression neutral?

• Is there evidence of unwanted motion in the capture?

• Is there evidence of interference (i.e. scalp hair) or artifacts that impact image quality?

• Is the image quality satisfactory?

• Is there adequate surface coverage for the targeted facial regions for the clinical or research study?

## Appendix 2. Summary of recommendations to optimize image acquisition

• Select a space with ample room for unobstructed flow and sufficient ambient lighting.

• Select seating that is appropriate for your population and will facilitate rapid positioning. When working with children, choose seating options that allow for maximum flexibility and safety.

• Prior to image capture, reposition any scalp hair that obscures relevant surface anatomy and remove all reflective objects.

• Work with the subject to achieve a "neutral" facial expression. If taking pre- and post-operative images, ask the subject to repeat his/her expression.

• To maximize facial surface coverage, position the patient's head so that priority areas are visible to the system's cameras or consider acquiring additional captures from alterative views.

• Consider batch processing when many images must be taken in a limited amount of time.

## Competing interests

The authors declare that they have no competing interests. The 3D images illustrated in this review were created with imaging systems designed by 3dMD (Atlanta, GA). The authors of this work do not have any financial disclosures or commercial associations with 3dMD or any other imaging device/company that might pose or create a conflict of interest with the information in this manuscript.

## Authors' contributions

CH and SW conceptualized the paper. CH, SW, KU and ES drafted and edited the manuscript.

All authors have read and approved the final manuscript.

## Authors' information

CH is affiliated with the Department of Pediatrics at the University of Washington, Seattle, WA. CH and ES are affiliated with the Children's Craniofacial Center at Seattle Children's Hospital, Seattle, WA. KU is affiliated with the Department of Epidemiology at the University of Washington. SW has a primary appointment at the Center for Craniofacial and Dental Genetics located within the Department of Oral Biology at the University of Pittsburgh, Pittsburgh, PA. SM also has secondary appointments in the Department of Anthropology and the Department of Orthodontics and Dentofacial Orthopedics at the University of Pittsburgh.
